# A versatile assay for RNA-binding proteins in living cells

**DOI:** 10.1261/rna.043562.113

**Published:** 2014-05

**Authors:** Claudia Strein, Anne-Marie Alleaume, Ulrich Rothbauer, Matthias W. Hentze, Alfredo Castello

**Affiliations:** 1 European Molecular Biology Laboratory (EMBL), 69117 Heidelberg, Germany; 2 Natural and Medical Science Institute at the University of Tuebingen, 72770 Reutlingen, Germany

**Keywords:** fluorescence, protein–RNA interaction, RNA, RNA-binding proteins

## Abstract

RNA-binding proteins (RBPs) control RNA fate from synthesis to decay. Since their cellular expression levels frequently do not reflect their in vivo activity, methods are needed to assess the steady state RNA-binding activity of RBPs as well as their responses to stimuli. While electrophoresis mobility shift assays (EMSA) have been used for such determinations, their results serve at best as proxies for the RBP activities in living cells. Here, we describe a quantitative dual fluorescence method to analyze protein–mRNA interactions in vivo. Known or candidate RBPs are fused to fluorescent proteins (eGFP, YFP), expressed in cells, cross-linked in vivo to RNA by ultraviolet light irradiation, and immunoprecipitated, after lysis, with a single chain antibody fragment directed against eGFP (GFP-binding protein, GBP). Polyadenylated RNA-binding activity of fusion proteins is assessed by hybridization with an oligo(DT) probe coupled with a red fluorophore. Since UV light is directly applied to living cells, the assay can be used to monitor dynamic changes in RNA-binding activities in response to biological or pharmacological stimuli. Notably, immunoprecipitation and hybridization can also be performed with commercially available GBP-coupled 96-well plates (GFP-multiTrap), allowing highly parallel RNA-binding measurements in a single experiment. Therefore, this method creates the possibility to conduct in vivo high-throughput RNA-binding assays. We believe that this fast and simple radioactivity-free method will find many useful applications in RNA biology.

## Abstract (precis)

This method paper describes a quantitative dual fluorescence approach to analyze protein–mRNA interactions in vivo.

## INTRODUCTION

Life depends on the coordinated temporal and spatial control of gene expression. Recent work has demonstrated that RNA levels only poorly correlate with protein abundance, implying that post-transcriptional regulation strongly contributes to sculpting the cellular proteome (Schwanhausser et al. 2011). Nascent, mature, and decaying messenger (m)RNAs assemble with RNA-binding proteins (RBPs) forming dynamic ribonucleoproteins (RNPs) that control mRNA fate (Glisovic et al. 2008). Both the RBP repertoire and the RBP activities of a given cell can respond to a multitude of biological cues and environmental stimuli, promoting the spatiotemporal regulation of gene expression by remodeling of RNPs (Gebauer and Hentze 2004; Muckenthaler et al. 2008; Martin and Ephrussi 2009; Mukhopadhyay et al. 2009). RBPs can bind mRNAs nonselectively with limited specificity or in a transcript-specific manner, organizing functionally related mRNAs into “regulons” by interacting with defined *cis*-regulatory elements (Gebauer and Hentze 2004; Keene 2007; Hentze et al. 2010). The complexity of protein–RNA networks and their regulation substantially increased by the recent discovery of hundreds of previously unidentified noncanonical RBPs using high-content approaches (Scherrer et al. 2010; Tsvetanova et al. 2010; Baltz et al. 2012; Castello et al. 2012; Klass et al. 2013; Kwon et al. 2013; Mitchell et al. 2013).

Ultraviolet (UV) cross-linking and immunoprecipitation (CLIP) followed by RNA sequencing or its modification using photoactivatable-ribonucleoside-enhanced cross-linking and immunoprecipitation (PAR-CLIP) have been successfully applied to identify RNAs as well as the exact RNA motifs recognized by RBPs in cultured cells (König et al. 2011a; Ascano et al. 2012). While CLIP benefits from the excitability of natural nucleic acid bases by 254-nm UV irradiation (conventional cross-linking cCL), PAR-CLIP employs the nucleotide analog 4-thiouridine (4-SU), which is taken up by cultured cells and incorporated into nascent RNAs. 4-SU is activated by 365-nm UV irradiation, driving efficient protein–RNA cross-linking (PAR-CL). In both cases, UV irradiation generates short-lived (μsec range) radicals that react with amino acids in close proximity (zero distance) forming covalent bonds (Pashev et al. 1991; Hafner et al. 2010a). In vivo UV cross-linking does not promote formation of covalent bonds between proteins (Pashev et al. 1991; Suchanek et al. 2005), and hence offers an approach to “freeze” directly binding RBPs to RNAs in vivo for subsequent immunoaffinity (König et al. 2011a; Ascano et al. 2012) or complementary nucleic acid-based isolation procedures (Baltz et al. 2012; Castello et al. 2012, 2013b; Gebauer et al. 2012).

The green fluorescent protein (GFP) and spectral variants thereof are widely used tools to study protein localization and dynamics in living cells (Chalfie et al. 1994; van Roessel and Brand 2002; Neumann et al. 2010). In spite of being the most commonly used protein tag in cell biology (Ghaemmaghami et al. 2003; Huh et al. 2003; Neumann et al. 2010), GFP is rarely applied to biochemical analyses. Recently a GFP-binding protein (GBP) derived from a single domain antibody from *Lama alpaca* has been described (Rothbauer et al. 2008). The GBP recognizes and binds with high-affinity wtGFP, eGFP, as well as variants of the yellow fluorescence protein YFP and eYFP (Rothbauer et al. 2008). GBP was successfully used for various biochemical applications, including studies of protein–protein and protein–DNA interactions or to modulate protein function in living cells (Rothbauer et al. 2008; Galan et al. 2011). Here, we present a fluorescence-based method that integrates UV irradiation-mediated in vivo cross-linking, GBP-based immunoprecipitation, and hybridization of co-isolated polyadenylated [poly(A)] RNAs with fluorescent oligo(DT) probes to quantify protein–RNA interactions taking place in cultured cells. While our standard protocol employs agarose-coupled GBP (GFP-Trap_A) in micro-tube format, the dual fluorescence RNA-binding assay can also be performed using GBP-coupled 96-well plates (GFP-multiTrap). Thus, multiple and highly parallel RNA-binding measurements (i.e., different proteins, conditions, controls, and biological replicates) can be performed in a single experiment, opening the possibility to quantify in vivo protein–RNA interactions in a high-throughput manner.

## RESULTS

### Analysis of RNA–protein interactions in living cells

**FIGURE 1.**
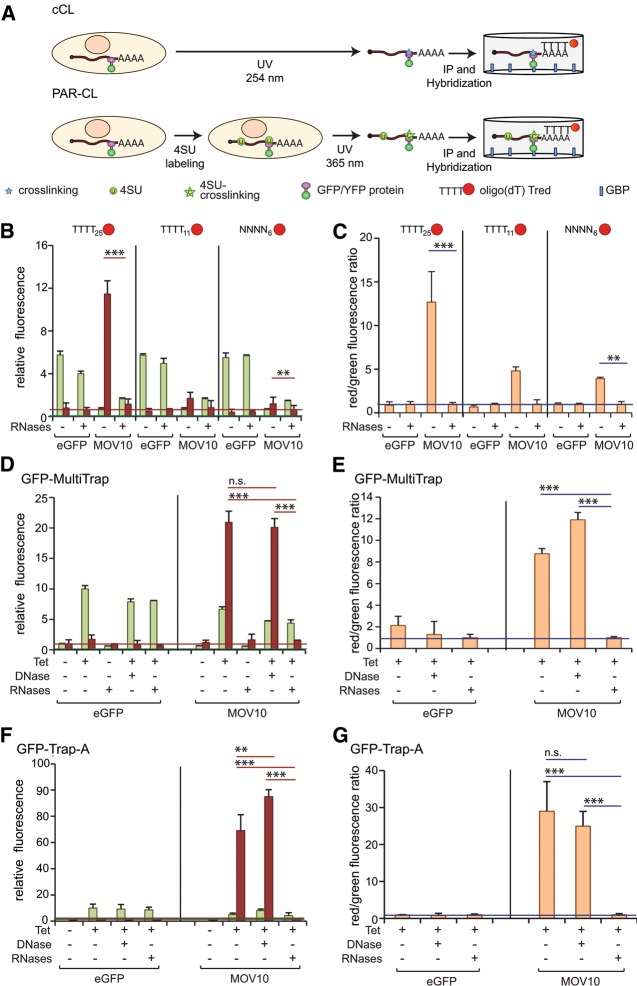
Quantitative assessment of protein–RNA interactions by the dual fluorescence RNA-binding assay. (*A*) Schematic representation of the dual fluorescence RNA-binding assay using conventional or PAR UV cross-linking protocols. (*B*,*C*) eGFP and MOV10–YFP cell lines were induced for 16 h and irradiated with 254-nm UV light. After lysis, control samples were treated with RNases. Recombinant proteins were immunoprecipitated using GFP-multiTrap. Co-immunoprecipitated RNAs were detected by hybridization with either 40 nM oligo(DT)_25_, oligo(DT)_11_, or random hexamers. The graphs represent relative green (eGFP/YFP) and red (Tred) fluorescence levels (*B*) and their ratios normalized to RNase-treated samples (*C*). (*D*,*E*) Dual fluorescence RNA-binding assay of tetracycline (Tet+) induced and noninduced (Tet−) Tet-on cell lines expressing eGFP or MOV10–YFP. Lysates were mock, RNase- or DNase-treated prior to immunoprecipitation in GFP-multiTrap. The graphs represent relative green (eGFP/YFP) and red (Tred) fluorescence levels (*D*), and their ratios normalized to RNase-treated samples (*E*). (*F*,*G*) As in *D* and *E*, but immunoprecipitation was carried out in microtubes using GFP-Trap_A. The graphs represent relative green (eGFP/YFP) and red (Tred) fluorescence levels (*F*) and their ratios normalized to RNase-treated samples (*G*). All graphs were generated from two biological and three technical replicates (total of six immunoprecipitations). (**) *P* < 0.01; (***) *P* < 0.001. Horizontal red and green lines represent the averaged Tred or eGFP background signals, respectively, extracted from control samples.

Current approaches to study and validate protein–RNA interactions in living cells use radiolabeled transfer to acceptor RNA (Baltz et al. 2012; Kwon et al. 2013). After UV cross-linking, RBPs are immunoprecipitated by specific antibodies and bound RNAs are detected by 5′ end labeling with ^32^P-γ-ATP phosphate and T4 polynucleotide kinase, followed by electrophoretic separation of labeled complexes. Using fluorescently labeled fusion proteins in combination with UV cross-linking to RNA, stringent GBP immunoprecipitation and oligo(DT) hybridization (Fig. 1A), we developed a robust method to measure the in vivo RNA-binding activities of RBPs (Castello et al. 2012). GFP- or YFP-tagged RBPs are expressed at physiological levels in a tetracycline (tet)-on inducible stable cell system (Flp-In TRex), in vivo cross-linked to their target RNAs by UV irradiation, and immunoprecipited with GBP. Co-isolated RNAs are subsequently hybridized with a fluorescent oligo(DT) probe (Fig. 1A). This method offers notable advantages over previously established protocols: (i) It does not require use of radioactivity; (ii) GBP is a highly selective binding molecule that allows efficient and specific immunoprecipitation of GFP(YFP)-tagged proteins under stringent conditions (Supplemental Fig. S1A–C), minimizing the co-purification of contaminants; (iii) the results can be obtained within a few hours simultaneously measuring the signals of the fluorescent fusion proteins and the fluorescently labeled oligo(DT) probe on a plate reader; (iv) the use of GBP-coupled 96-well plates (GFP-multiTrap) allows experimental scale up to high-throughput conditions.

We first examined the efficiency and specificity of Texas red (Tred)-labeled oligo(DT)_25_, oligo(DT)_11_, and random hexamers to detect polyadenylated RNA in samples from cells expressing the broad specificity RBP MOV10 fused to YFP (Castello et al. 2012). As a negative control we used cells expressing unfused eGFP, which only differs in four amino acids from YFP. Both proteins were immunoprecipitated with GFP-multiTrap using stringent conditions and RNA was subsequently quantified by hybridization with the different probes. Maximal absorption and emission of eGFP and YFP slightly differ; we therefore optimized these parameters for both fluorescent proteins. For simplicity, we will refer to the fluorescence emitted by either of the two proteins as “green fluorescence.” MOV10–YFP samples showed substantially higher red fluorescence (i.e., probe binding) as well as red/green (RNA/protein) fluorescence ratio with all probes than eGFP control immunoprecipitations (Fig. 1B,C). Nevertheless, the oligo(DT)-derived signal was significantly stronger with oligo(DT)_25_ than with oligo(DT)_11_ and random hexamers (Fig. 1B,C). The low signal to noise ratio of oligo(DT)_11_ suggests that long poly(A)/oligo(DT) sequences are required for efficient hybridization under our experimental conditions. Such long poly(A) tracts are rarely found in internal regions of cellular RNAs, but are typically present at the 3′ end of polyadenylated RNAs. As an additional control, half of the samples were treated with RNases to assess whether the oligo(DT)-derived signal is RNase-sensitive. RNase treatment strongly diminished the Tred signal without affecting YFP fluorescence when an oligo(DT)_25_ probe was used (Fig. 1B,C). In contrast, the impact of RNase treatment on oligo(DT)_11_ or random hexamer-derived fluorescence was more modest, reflecting the lower signal to noise ratio of these probes (Fig. 1B,C). Of relevance, neither Tred nor GFP fluorescence was affected by DNase treatment (Fig. 1D,E), further supporting the specificity of oligo(DT)–poly(A) RNA hybridization under our experimental conditions. As expected, the induction of MOV10–YFP is necessary to detect a significant Tred signal over background, since only marginal green and red fluorescent signals were detected from noninduced (Fig. 1D,E) and parental (data not shown) HeLa Flp-In TRex cells. Taken together, all these results highlight oligo(DT)_25_ as the best probe for the specific detection of polyadenylated RNA. Furthermore, we noticed that the normalization of the red/green fluorescence ratios from untreated to RNase-treated samples adds an informative specificity parameter.

Notably, the red/green ratio for MOV10 was about three times higher with GFP-Trap_A than with GFP-multiTrap (Fig. 1, cf. D,E and F,G). These results are probably due to the higher binding capacity of GFP-Trap_A over GFP-multiTrap. One well of the GFP-multiTrap binds 0.5–1 µg of purified GFP, which is comparable to 1 µL slurry of GFP-Trap_A. Since we use 10 μL of GFP-Trap_A (5–10 μg binding capacity) in our in-tube settings, we recommend the use of this capture reagent to study RBPs that are expressed at modest levels or that display low UV cross-linking efficiencies.

**FIGURE 2.**
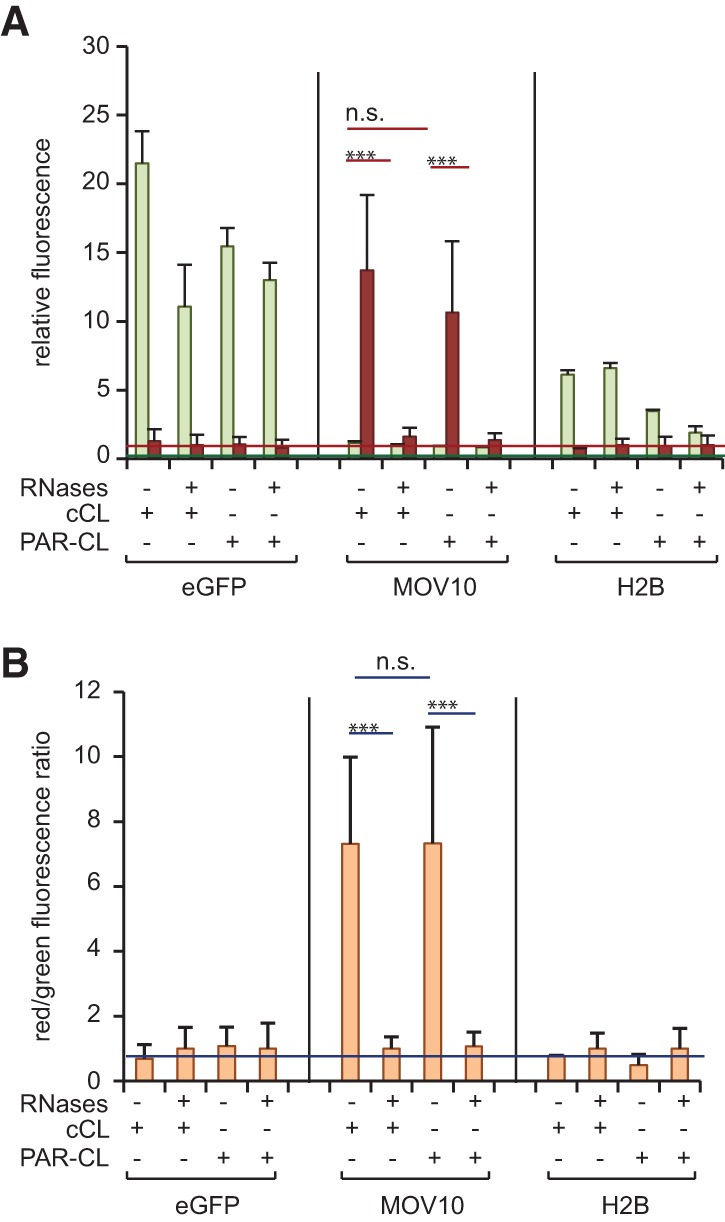
Both cCL and PAR-CL protocols can be applied to the dual fluorescence RNA-binding assay. Comparison of the performance of cCL and PAR-CL in the dual fluorescence RNA-binding assay. eGFP, MOV10–YFP, and H2B–eGFP cell lines were induced for 16 h. Protein–RNA complexes were cross-linked applying cCL or PAR-CL. After lysis, control samples were RNase-treated and immunoprecipitated using GFP-multiTrap. The graphs show relative green (eGFP/YFP) and red (Tred) fluorescence levels (*A*) and their ratios normalized to RNase-treated samples (*B*). The graphs were generated from two biological and three technical replicates (total of six immunoprecipitations). (***) *P* < 0.001. Horizontal red and green lines represent averaged Tred or eGFP background signal, respectively, extracted from control samples.

Due to differences in the physicochemical properties of cCL and PAR-CL, some RBPs display higher cross-linking efficiencies with one approach compared with the other. In particular, 24% and 12% of the HeLa mRNA interactome proteins are favored by either cCL or PAR-CL, respectively (Castello et al. 2012). Thus, to allow the study of the maximal spectrum of RBPs with the dual fluorescence RNA-binding assay, we also explored the use of PAR-CL. Because MOV10 displays similar cross-linking efficiencies to RNA with cCL and PAR-CL (Castello et al. 2012), we used the MOV10–YFP cell line to assess the performance of both cross-linking procedures in the dual fluorescence RNA-binding assay. eGFP and the DNA-binding protein Histone-2B (H2B) fused to eGFP were employed as negative controls. After overnight incubation of cultured cells with or without 4-SU, cell monolayers were irradiated with either 365-nm or 254-nm UV light, respectively, as outlined in Figure 1A (Castello et al. 2013b). MOV10 immunoprecipitation with GFP-multiTrap yielded similar green and red fluorescence irrespective of the UV cross-linking protocol applied (Fig. 2A). As a consequence, the RNA/protein ratio from MOV10 samples is almost identical following either of the two protocols (Fig. 2B). Irrespective of the cross-linking method, red fluorescence from MOV10 samples is sensitive to RNase treatment and significantly higher than that of the two negative controls. Therefore, the dual fluorescence RNA-binding assay is compatible with both cCL and PAR-CL.

### Enhancing signal-to-noise ratios by oligo(DT)_25_ probe modifications

**FIGURE 3.**
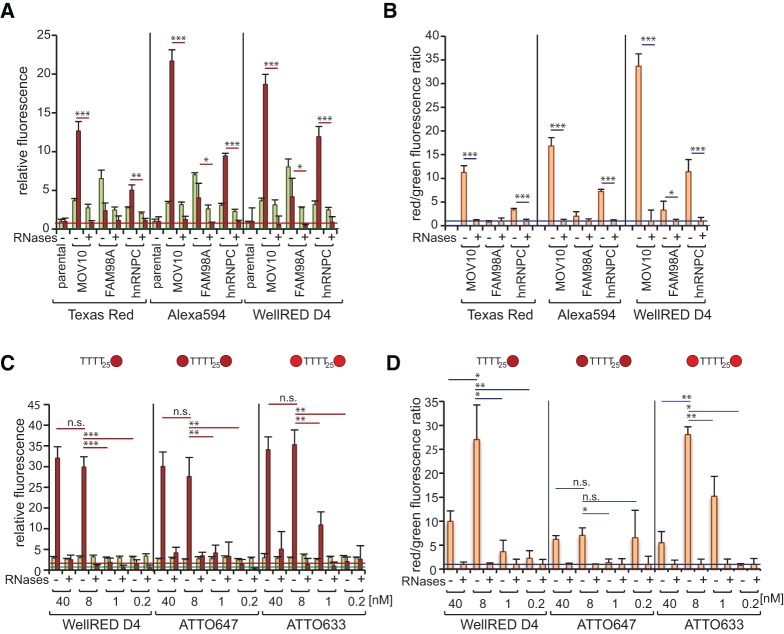
Evaluation of different fluorophores and probes in the dual fluorescence RNA-binding assay using GFP-multiTrap. (*A*,*B*) Parental, MOV10–YFP, hnRNPC–eGFP, and FAM98A–eGFP cell lines were employed for the dual fluorescence RNA-binding assay using oligo(DT)_25_ probes coupled with Tred, Alexa Fluor594, or WellRED D4. After 254-nm UV irradiation and cell lysis, control samples were RNase-treated prior to immunoprecipitation in GFP-multiTrap. The graphs represent relative green (eGFP/YFP) and red (Tred/Alexa Fluor594/WellRED D4) fluorescence levels (*A*) and their ratios normalized to RNase-treated samples (*B*). The graphs were generated from two biological and three technical replicates (total of six immunoprecipitations). (*C*,*D*) MOV10–YFP was induced for 16 h and cells were irradiated with 254-nm UV light. Control samples were RNase-treated and fluorescent fusion proteins were immunoprecipitated with GFP-multiTrap. Co-immunoprecipitated RNAs were hybridized with 40, 8, 1 or 0.2 nM oligo(DT) probe coupled with either one WellRED D4 molecule or two ATTO 647 or ATTO633 molecules. The graphs represent relative green (YFP) and far red (WellRED D4/ATTO 647/ATTO633) fluorescence levels (*C*) and their ratios normalized to RNase-treated samples (*D*). The graphs were generated from three technical replicates (total of three immunoprecipitations). (*) *P* < 0.05; (**) *P* < 0.01; (***) *P* < 0.001. Horizontal red and green lines represent averaged background signal for red and green signal, respectively, extracted from control samples.

Family-with-sequence-similarity (FAM)98A has recently been identified and validated as an RBP in two independent studies (Baltz et al. 2012; Castello et al. 2012). While FAM98A displayed significant RNA-binding activity in the dual-fluorescence RNA-binding assay after pulldown with the GFP-trap_A (Castello et al. 2012), it suffered from a low signal to noise ratio with GFP-multiTrap (Fig. 3A,B). Even the heterogeneous nuclear ribonucleoprotein C (hnRNPC) only yielded a modest Tred signal (Fig. 3A,B). Thus, we attempted to enhance the RNA-derived signal in GFP-multiTrap immunoprecipitations by using probes coupled with brighter fluorophores. Oligo(DT)_25_ labeled with Alexa Fluor 594 was chosen for its extinction coefficient twofold higher than that of Tred. Since emission of naturally fluorescent molecules does not reach the far-red spectrum, WellRED D4 with an emission peak at 670 nm may reduce the background signal and was therefore selected as an alternative fluorophore for the Oligo(DT)_25_ probe. The alexa Fluor 594 probe showed a modest improvement of the red/green fluorescence ratio of MOV10 and hnRNPC samples over RNase-treated controls, while the RNA-binding activity of FAM98A remained undetectable under these conditions in GFP-multiTrap assays (Fig. 3A,B). Strikingly, the WellRED D4-labeled probe enhanced the RNA-derived signal from all RBP samples tested about threefold, including FAM98A. Indeed, the red/green fluorescence ratio of untreated FAM98A samples was significantly higher than that of the RNase-treated counterparts (Fig. 3A,B). This substantial improvement in sensitivity makes on-plate dual fluorescence RNA-binding assays feasible for proteins with limited RNA-binding activity/UV cross-linking efficiency.

An additional option to enhance the RNA-derived signal is to include more than one fluorophore per oligo(DT) probe. As the chemistry of WellRED D4 is incompatible with multiple labeling, we tested the performance of oligo(DT)_25_ probes labeled with two molecules of ATTO647 or ATTO633. These fluorophores can be coupled simultaneously with the 5′ and 3′ ends of the DNA probe and display comparable absorption and emission wavelengths as well as extinction coefficients to WellRED D4. Since the two fluorophores are separated by 25 nt, quenching of the fluorescent signal is minimal. The optimal concentration of the Tred probes was determined to be 40 nM (data not shown). Because the optimal concentration of the doubly labeled probes may differ from that of the singly labeled ones, we conducted a titration experiment. The optimal red/green fluorescence ratio was obtained at 8 nM for all three far red probes, probably due to a lower basal red fluorescence in negative controls (Fig. 3C). The MOV10 red/green fluorescence ratio was similar for the WellRED D4 and ATTO633 probes, whereas the ATTO 647 probe yielded inferior results (Fig. 3C,D). ATTO647 has a higher hydrophobicity coefficient than its counterpart ATTO633, which may explain the higher basal signal due to possible nonspecific interactions of the fluorophore with hydrophobic amino acids or nucleotide bases (Fig. 3C,D). However, both WellRED and the doubly labeled ATTO633 probes perform similarly at 8 nM, improving the signal in comparison to TRed probes by ∼2.5-fold in both cases (Fig. 3, cf. B and D). Both probes appear to be equally suitable for on-plate high-throughput experiments.

### Applications of the dual fluorescence RNA-binding assay

#### Validation of candidate RNA-binding proteins

**FIGURE 4.**
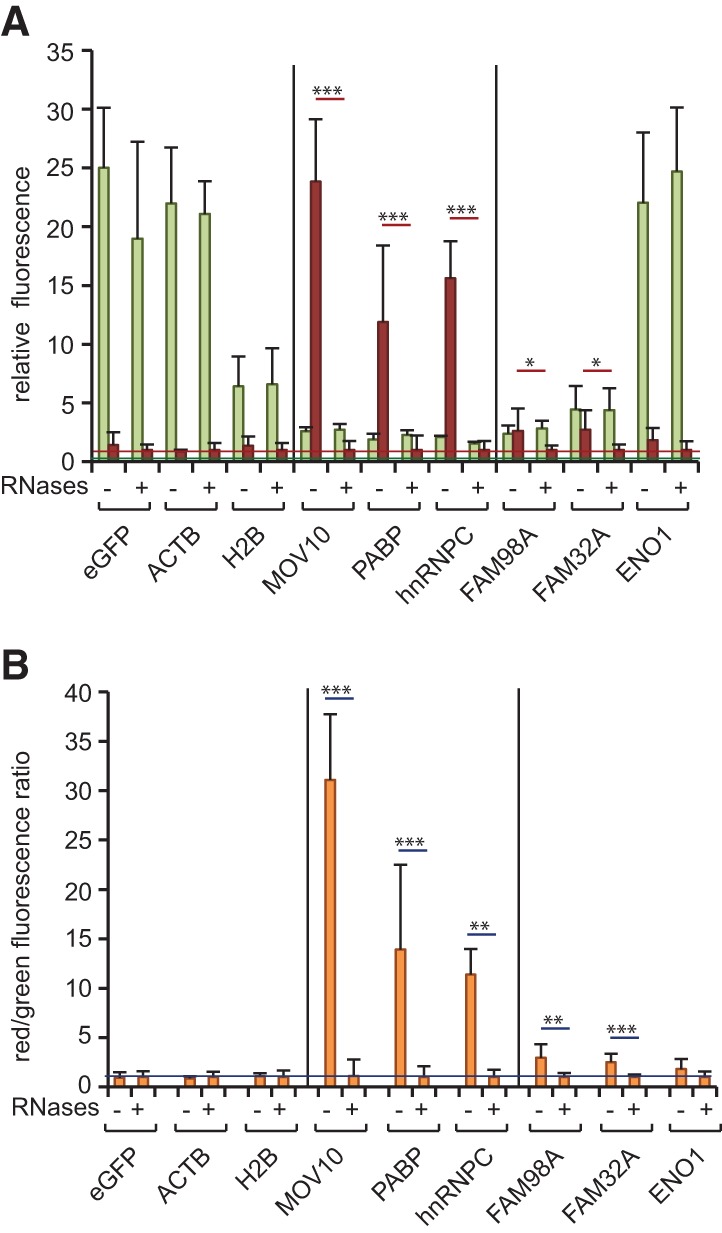
Application examples of the dual fluorescence RNA-binding assay. (*A*,*B*) Cells expressing eGFP, ACTB–eGFP, H2B–eGFP, MOV10–YFP, hnRNPC–eGFP, PABP–eGFP, FAM98A–eGFP, FAM32A–eGFP, or ENO1–YFP were induced with tetracycline for 16 h and irradiated with 254-nm UV light. After lysis, control samples were RNase-treated and recombinant proteins were immunoprecipited with GFP-multiTrap. Co-immunoprecipitated RNAs were hybridized with oligo(DT) coupled with one molecule of WellRED D4. The graphs represent relative green (eGFP/YFP) and far red (WellRED D4) fluorescence levels (*A*) and their ratios normalized to RNase-treated samples (*B*). The graphs were generated from three biological and three technical replicates (total of nine immunoprecipitations). (*) *P* < 0.05; (**) *P* < 0.01; (***) *P* < 0.001. Horizontal red and green lines represent averaged eGFP or Tred background signal, respectively, extracted from control samples.

The dual fluorescence RNA-binding assay described here can be used to validate the in vivo RNA-binding activity of candidate RBPs identified by system-wide approaches (Scherrer et al. 2010; Tsvetanova et al. 2010; Baltz et al. 2012; Castello et al. 2012; Klass et al. 2013; Kwon et al. 2013; Mitchell et al. 2013). We assessed the RNA-binding activity of three previously known RBPs (MOV10, hnRNPC, and cytoplasmic poly(A)-binding protein [PABP]), three recently discovered RBPs (FAM98A, FAM32A, and enolase 1 [ENO1]), and three negative controls (H2B, β-actin [ACTB], eGFP). The three classical RBPs show high red/green fluorescence ratios in untreated compared with RNase-treated samples (Fig. 4A,B). Two (FAM98A, FAM32A) out of the three recently discovered RBPs show a modest but significant RNA-derived signal, which is sensitive to RNase treatment (Fig. 4A,B). FAM98A localizes in nuclear speckles, whereas FAM32A mainly has a nucleolar distribution (Supplemental Fig. S2). Their RNA-binding activity and localization to RNA-rich nuclear structures strongly supports their role in RNA metabolism. Although the red signal is higher in untreated than in RNase-treated ENO1 samples, this difference does not reach statistical significance (Fig. 4A,B). ENO1 has been validated as an RBP using GFP-Trap_A as well as CLIP followed by next-generation sequencing (Castello et al. 2012). The low signal to noise ratio of ENO1 reflects potential limitations regarding the sensitivity of the GFP-multiTrap-based assay for RBPs with limited UV cross-linking efficiency.

#### Mapping the RNA-binding domains of RBPs

**FIGURE 5.**
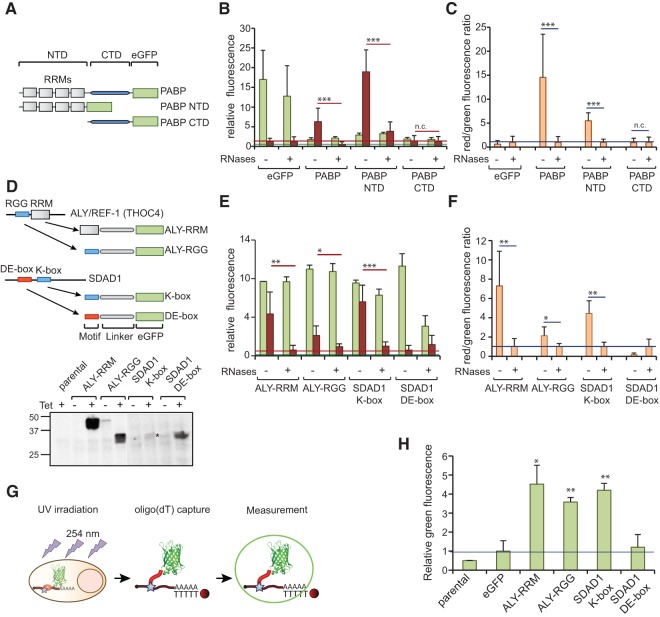
Studying RNA-binding domains by dual fluorescence RNA-binding assay. (*A*) Schematic representation of the PABP–eGFP fusion protein and its deletion mutants. (*B*,*C*) eGFP, PABP–eGFP, PABP–NTD–eGFP, and PABP–CTD–eGFP cell lines were induced for 16 h and irradiated with 254-nm UV light. Control samples were RNase-treated and recombinant proteins were immunoprecipitated with GFP-multiTrap. Co-immunoprecipitated RNAs were hybridized with oligo(DT) coupled with WellRED D4. The graphs represent relative green (eGFP) and far red (WellRED D4) fluorescence levels (*B*) and their ratios normalized to RNase-treated samples (*C*). The graphs were generated from three biological and three technical replicates (total of nine immunoprecipitations). (*D*) Schematic representation of the eGFP-fused RBP motifs of ALY and SDAD1. After incubation of the respective cell lines for 16 h with or without tetracycline, cells were lysed in loading buffer. Expression levels and molecular size of eGFP fusion proteins were determined by Western blotting. (*E*,*F*) The eGFP fusion proteins were induced in HeLa Flp-In cells for 16 h by addition of tetracyclin. After lysis, control samples were RNase-treated and recombinant proteins were immunoprecipitated with GFP-multiTrap. Co-immunoprecipitated RNAs were hybridized with oligo(DT) coupled with WellRED D4. The graphs represent relative green (eGFP) and far red (WellRED D4) fluorescence levels (*E*) and their ratio normalized to RNase-treated samples (*F*). The graphs were generated from two biological and three technical replicates (total of six immunoprecipitations). (*G*) Schematic representation of interactome capture. (*H*) eGFP fusion proteins were expressed in HeLa Flp-In cells for 16 h and irradiated with 254-nm UV light. After lysis, protein–RNA complexes were isolated with oligo(DT) and eluted in a low salt buffer at 55°C. Green fluorescence (eGFP) was measured in a plate reader and represented in a graph. (*) *P* < 0.05; (**) *P* < 0.01; (***) *P* < 0.001. Horizontal red and green lines represent averaged eGFP or Tred background signal, respectively, extracted from control samples.

PABP interacts with poly(A) tracts by means of four RNA-recognition motifs (RRM) located in its N-terminal domain (NTD) (Fig. 5A), whereas its C-terminal domain (CTD) mediates other biological functions such as protein–protein interactions (Kahvejian et al. 2005; Lloyd 2006). To determine whether the dual fluorescence RNA-binding assay can be employed to define the RNA-binding region of a given RBP, we analyzed the ability of full-length PABP, its NTD, or its CTD to bind RNA. All three GFP fusion proteins are immunoprecipitated with similar efficiency; however, only PABP and –NTD yield significant red fluorescence compared with their respective RNase-treated controls (Fig. 5B,C), and only the red/green fluorescence ratios of untreated PABP and –NTD samples are significantly higher than their RNase-treated controls. Moreover, the insignificant red fluorescence displayed by PABP–CTD samples is insensitive to RNase treatment. Our results clearly indicate that the RNA-binding moiety of PABP is located within its four tandem RRM-containing NTDs, in agreement with previous reports (Deo et al. 1999; Safaee et al. 2012).

Analysis of the HeLa mRNA interactome revealed that intrinsically disordered regions are overrepresented within RBPs and that their amino acids cluster into repetitive motifs. Combination of arginine (R) with glycine (G), referred to as RGG-box, is a classical RBP motif with a well-defined role as RNA-binding domain (RBD) (Phan et al. 2011; Thandapani et al. 2013). Different reports propose that RGG-boxes cooperate with globular RBDs to increase the binding affinity and specificity of an RBP to its target RNA (Thandapani et al. 2013). We assessed whether the RGG-box and the RRM of the RNA export factor ALY (also known as REF1 and THOC4; see Fig. 5D) can be identified as individual and independently functional RNA-binding modules. We established HeLa cell lines expressing either region as a GFP fusion protein (Fig. 5D). Both the RGG-box and RRM yield significant RNA-derived signals sensitive to RNase treatment. The red/green fluorescence ratio of ALY RRM samples is sevenfold higher than that of the corresponding samples treated with RNases (Fig. 5E,F). The ALY RGG-box by itself shows a modest (approximately twofold over background) but significant RNA-derived signal. To substantiate these findings, we applied the interactome capture protocol to these cell lines (Castello et al. 2013b). Cell monolayers were irradiated with 254-nm UV light to covalently cross-link RBPs to RNA. After lysis, protein–RNA complexes are isolated via oligo(DT) pull down. The amount of eGFP-tagged protein present in eluates is detected by fluorescence measurement on a plate reader (Fig. 5G). In agreement with the dual fluorescence RNA-binding assay, the RRM and RGG-box of ALY are both efficiently isolated by interactome capture, whereas the fluorescence detected in eGFP control samples is close to background, as defined by the parental cell line (Fig. 5H). Taken together, these results indicate that both the ALY RRM and RGG-box can function as RNA-binding modules in living cells.

Furthermore, mRNA interactome capture had identified RBPs with long and repetitive basic patches rich in lysines (K), named K-boxes, which were found to be especially abundant among newly identified RBPs (Castello et al. 2012). Previous reports showed that disordered basic tracts can mediate DNA-binding in homeodomain transcription factors, promoting DNA scanning toward their specific binding sites through a linear diffusion mechanism (Iwahara and Clore 2006; Iwahara et al. 2006; Vuzman et al. 2010; Vuzman and Levy 2010). However, it is unknown whether K-boxes can fulfill a similar function for RBPs. Interestingly, K-boxes often co-occur and, in some instances, alternate with acidic patches (Castello et al. 2012). The role of the co-occurrence of acidic and basic patches is still unknown, but these patches may promote intra- or intermolecular interactions. To determine whether the K-box of SDAD1 can mediate RNA binding, we fused it to eGFP. This basic patch is composed of 45 amino acids, including 15 lysines, one arginine, two glutamines and two asparagines (N), amino acids that are frequently found at protein–RNA interfaces (Lunde et al. 2007). As a control we expressed the SDAD1 acidic patch (DE-box), which is adjacent to the K-box (Fig. 5D). This disordered region of 33 amino acids harbors seven aspartic acids and five glutamic acids. In spite of the presence of two lysines and one arginine, we did not expect this acidic polypeptide to bind to RNA. Both eGFP fusion proteins localized mainly to the nuclei of induced cells (Fig. 5D). Interestingly, the K-box but not the DE-box yield significantly higher red fluorescence in untreated than in RNase-treated samples (Fig. 5E). To confirm the RNA-binding properties of the SDAD1 K-box, we also performed an interactome capture experiment (see above). Green fluorescence strongly and significantly exceeds the background when the assay was performed with the K-box–eGFP cell line, but not with DE-box–eGFP (Fig. 5H). Thus, long basic disordered patches can mediate interactions of a protein with poly(A) RNA in cultured cells, which could contribute to the overall RNA-binding affinity of a given RBP. These examples demonstrate the utility of the dual fluorescence RNA-binding assay for the characterization of RBDs using protein mutants (Fig. 5C) or the individual expression of short protein motifs (Fig. 5F).

## DISCUSSION

Biochemical in vitro approaches, such as the electrophoretic mobility shift assay (EMSA), have been successfully applied to study RBPs. However, in a biological context the RNA-binding activity of RBPs is typically regulated by a plethora of mechanisms, including post-translational modifications (Mukhopadhyay et al. 2008; Arif et al. 2009), induced conformational changes (Walden et al. 2006; Siddiqui et al. 2012), competition with other RBPs for target RNAs (Yao et al. 2012), differential subcellular localization (Alvarez et al. 2013), alterations of its expression levels, or the availability of its target RNAs. Importantly, most of the proteins within the HeLa mRNA interactome are post-translationally modified (http://www.embl.de/mRNAinteractome) (Castello et al. 2012), suggesting that RBPs may globally respond to environmental alterations. As an illustrative example, glutamyl-prolyl tRNA synthetase (EPRS) is phosphorylated in macrophages in response to interferon-γ treatment, triggering its release from the multi-synthetase complex and its subsequent assembly into the IFN-γ-activated inhibitor of translation (GAIT) complex (Mukhopadhyay et al. 2009). Phosphorylation also promotes conformational changes in the WHEP domains of EPRS that activate its mRNA-binding activity, enabling translational repression of proinflammatory genes (Jia et al. 2008; Arif et al. 2009, 2011). The REM (RNA, enzyme, and metabolite) network hypothesis (Hentze and Preiss 2010) proposes an additional layer of RNA-binding regulation, where substrate (or cofactor) and RNA compete for the metabolite-binding pocket of an enzyme. In this context, the metabolic and RNA-binding activity of these moonlighting enzymes may be modulated by metabolite availability in addition to post-translational modifications. This form of RBP regulation is exemplified by iron regulatory protein 1, which switches from metabolic to RNA-binding function when it cannot assemble with [Fe-S] cluster, a cofactor necessary for its catalytic activity (Walden et al. 2006; Muckenthaler et al. 2008). In other instances, mutations in RBPs are associated with human diseases, such as neurological disorders, muscular atrophies, or cancer (Lukong et al. 2008; Darnell 2010; Castello et al. 2013a). Interestingly, disease mutations often map to the RNA-binding domains and disordered regions of RBPs, although the functional impact of these mutations on RNA-binding remains to be assessed in most cases (Castello et al. 2013a). Thus, there is a need for methods to assess protein–RNA interactions in a physiological context.

Users of the dual fluorescent assay need to consider that the approach will fail to detect physiological RBP–RNA interactions when (i) protein-bound RNAs are not polyadenylated; (ii) the RBP fails to cross-link efficiently by either cCL or PAR-CL; (iii) the eGFP tag interferes with protein folding or RNA binding; (iv) target RNAs are expressed at low levels and the ectopically expressed RBP has to compete with its endogenous counterpart.

Nevertheless, the method reported here has notable advantages over existing methodologies: (i) UV light is directly applied to monolayers of living cells, thus “freezing” physiological in vivo protein–RNA interactions; (ii) UV irradiation induces covalent bond formation between RBPs and RNA, but unlike chemical cross-linking does not promote protein–protein covalent bonds, allowing the specific capture of direct RNA binders following an immunoprecipitation procedure; (iii) GBP allows the high affinity and specificity isolation of eGFP- and YFP-tagged proteins, which is compatible with the use of high salt buffers (up to 1 M NaCl) and ionic detergents (up to 0.1% SDS), leading to efficient removal of noncovalently associated proteins; (iv) this assay is based on nonhazardous, rapid fluorescence measurements and the RNA-derived signal can be easily normalized to the fluorescence of immunoprecipitated GFP/YFP providing an accurate and reliable readout; (v) the dual fluorescence RNA-binding assay can be performed using commercially available GBP-coupled 96-well plates (GFP-multiTrap). GFP-multiTrap allows 96 immunoprecipitations simultaneously, decreasing the technical variability of the assay and enabling high-throughput applications. This important feature now allows studying the RNA-binding activity of a given RBP quantitatively under a dozen of different experimental conditions, even including technical and biological replicates as well as appropriate quality controls within the same plate.

The dual fluorescence RNA-binding assay allows identification of RNA-binding domains and motifs. Here, we showed that the RBD of PABP is harbored within its RRM-containing NTD, whereas its CTD lacks RNA-binding activity. These in vivo data reflect previous structural information (Deo et al. 1999; Safaee et al. 2012) and support the differential role of NTD and CTD in PABP function. Importantly, we showed that short disordered and repetitive motifs, such as RGG-boxes, can endow eGFP with RNA-binding activity, indicating that these disordered motifs play important roles in RBP function. Lysine-rich motifs are frequently found in RBPs, representing nuclear localization signals (NLS) in some cases but often being longer than canonical NLS. These motifs frequently co-occur with other repetitive motifs (e.g., acidic patches) and flack globular domains (Castello et al. 2012). The dual fluorescence RNA-binding assay revealed that the SDAD1 K-box can indeed mediate strong RNA-binding activity. Aligned lysine motifs may establish electrostatic interactions with the phosphate backbone, as previously described for basic tails in homeodomain-containing transcription factors and their interaction with DNA (Iwahara et al. 2006). Therefore, the dual fluorescence RNA-binding assay can be employed to define the biological activity of short RBP motifs and the implications of post-translational modifications and disease-associated mutations in their potential RNA-binding activity.

### Perspectives

The dual fluorescence RNA-binding assay described in this report complements existing methodologies such as CLIP (König et al. 2011a; Ascano et al. 2012) or interactome capture (Castello et al. 2013b), representing a tool to validate protein–RNA interactions. Apart from this application, the quantitative properties of the assay can help to generate informative RNA-binding activity parameters for individual RBPs in different cell types, environmental conditions (e.g., stress, infection, starvation), or biological systems (e.g., *Saccharomyces cerevisiae*, *Caenorhabditis elegans*, *Drosophila* embryos), providing a valuable resource to study RBP dynamics. Importantly, GFP-multiTrap allows highly parallel RNA-binding quantifications in a single experiment, offering the possibility to investigate the dynamics of RBPs under different environmental conditions. The method can also be used to map and characterize the RNA-binding architectures of RBPs in living cells, including disordered protein motifs of unknown function. The high-throughput potential of the dual-fluorescence RNA-binding assay may also help with exhaustive mutagenesis analyses to determine the role of individual amino acids (e.g., disease-associated mutations) in RNA-binding or with small molecule screening projects.

## MATERIALS AND METHODS

### Generation of stable cell lines

Chimeric cDNAs encoding the different eGFP- or YFP-tagged proteins were amplified by PCR from already established eGFP/YFP-containing plasmid libraries (Castello et al. 2012). Alternatively, a HeLa cDNA library and eGFP plasmid were used as templates for fusion PCR. Resulting chimeric cDNAs were cloned into pCDNA5/FRT/TO (Life Technologies). HeLa cell lines were established as described in the manufacturer's protocols (Flp-In TRex, Life Technologies).

### The dual fluorescence RNA-binding assay: in-tube settings

For cCL, one 10-cm dish of HeLa cells at 40%–50% confluence was incubated for 16 h at 37°C with DMEM supplemented with 5% fetal calf serum (FCS) and 1 μg/mL tetracycline to induce the expression of the eGFP- or YFP-tagged proteins. Next, cells were washed twice with 5 mL of PBS. After complete removal of the PBS, cells were placed on ice and immediately irradiated with 0.15 J/cm^2^ UV light at 254 nm (König et al. 2011b; Castello et al. 2013b). For PAR-CL, cells were incubated for 16 h with DMEM supplemented with 5% FCS, 1 μg/mL tetracycline, and 100 μM of 4-SU. After two washes with PBS, protein–RNA cross-linking was accomplished by irradiation with 0.15 J/cm^2^ UV light at 365 nm as previously described (Hafner et al. 2010b; Castello et al. 2013b). Cells were scraped into 5 mL of ice-cold PBS, collected by centrifugation, and resuspended in 300 μL of lysis buffer (100 mM KCl, 5 mM MgCl_2_, 10 mM Tris pH 7.5, 1% NP40, 1 mM DTT, 100 units/mL RNAseOUT [Life Technologies], 1× protease inhibitor cocktail [Roche], and 200 μM ribonucleoside vanadyl complex [NEB]). Lysates were incubated for 10 min at 4°C, snap-frozen, and thawed, to accomplish complete cell lysis. Lysates were centrifuged for 10 min at 10,000 rpm and 4°C to pellet cellular debris, and the supernatants were collected and aliquoted into three tubes (100 μL per tube) for technical triplicates. Samples were mixed with 400 μL of dilution buffer (500 mM NaCl, 1 mM Mg_2_Cl, 0.05% SDS, 0.05% NP-40, 50 mM Tris-HCl pH 7.5), and then supplemented with 100 units/mL RNaseOUT, 1× protease inhibitor cocktail (Roche), and 10 μL of pre-equilibrated GFP-Trap_A (Chromotek). GFP-tagged proteins were immunoprecipitated for 2 h at 4°C with gentle rotation, subsequently washed with 500 μL of medium salt buffer (250 mM NaCl, 1 mM Mg_2_Cl, 0.025% SDS, 0.05% NP-40, 20 mM Tris-HCl pH7.5), and incubated for 15 min at 4°C with 250 μL of blocking solution (200 mM LiCl, 20 mM Tris pH 7.5, 1 mM EDTA, 0.01% NP40, 100 μg/mL *Escherichia coli* tRNA, 100 μg/mL BSA). Hybridization was performed by incubation with 250 μL of hybrydization buffer (500 mM LiCl, 20 mM Tris pH 7.5, 0.05% LiDS, 1 mM EDTA, 5 mM DTT, 0.01% NP40, 100 units/mL RNaseOUT) supplemented with 40 nM of oligo(DT)_25_-Tred (Sigma) for 1 h at 4°C. Excess of fluorescent probe was removed by washing once with 500 μL of wash buffer 1 (500 mM LiCl, 20 mM Tris pH 7.5, 0.01% LiDS, 0.01% NP40, 1 mM EDTA, 5 mM DTT) and twice with 500 μL of wash buffer 2 (200 mM LiCl, 20 mM Tris pH 7.5, 0.01% LiDS, 0.01% NP40, 1 mM EDTA, 5 mM DTT). GFP-Trap_A was resuspended in 100 μL of the latter buffer and transferred to a 96-well optical plate (CBG Thermo scientific, NUNC). All buffers indicated above were supplemented with 50 units/mL of RNaseOUT and 1× protease inhibitor cocktail. For control RNase treatments see below.

### On plate dual fluorescence RNA-binding assay

Cells were grown, UV-irradiated and recovered as described above, and then lysed into 300 μL of lysis buffer. Lysates were centrifuged for 10 min at 10,000 rpm and 4°C to pellet cellular debris. The supernatants were collected, their volumes adjusted to 400 µL with lysis buffer, and then supplemented with 100 μL of 5× dilution buffer (1.25 M NaCl, 100 mM Tris-HCl pH7.5) and 5 µL 5% SDS, and transferred to three wells (150 μL per well) of the GFP-mutiTrap (Chromotek) for technical triplicates. Immunoprecipitation was performed overnight at 4°C with agitation (800 rpm). We recommend distributing the technical replicates to distant positions from each other on the plate to control for reading and immunoprecipitation homogeneity. Samples were washed with 180 μL of medium salt buffer and then incubated for 15 min with 180 μL of blocking solution at 4°C. Hybridization was performed with 100 µL hybridization buffer supplemented with 40 nM oligo(DT)_25_ Tred/8 nM of oligo(DT)_25_-WellRED for 1 h at 4°C. Excess of fluorescent probe was removed by washing once with wash buffer 1 and twice with wash buffer 2. Finally, 100 μL of wash buffer 2 was added to each well and fluorescence was immediately measured as described below.

### Fluorescence measurements

Fluorescence was measured in a TECAN Safire II microplate reader, using the following parameters: eGFP/YFP: the optimal excitation and emission are 475 nm and 509 nm, respectively, for eGFP and 514 nm and 527 nm for YFP. We determined that excitation 490 nm, emission 515 nm recovered comparable signals from both proteins. These parameters were used for experiments monitoring simultaneously both eGFP and YFP. For red and far red fluorophores we applied the following parameters: Tred and Alexa Fluor594 excitation 590 nm, emission 616 nm; WellRED and ATTO 647: excitation 650 nm, emission 670 nm. ATTO633: excitation 630 nm, emission 657 nm. In all cases bandwidth was restricted to 5–10 nm and gain was set using the positive controls as a reference. For higher reliability, we recommend measuring from the bottom of the plate (when this option is available).

### RNase and DNase treatment

Cell lysis was performed in lysis buffer lacking RNase inhibitors (i.e., ribonucleoside vanadyl complex and RNaseOUT). Lysates were incubated for 10 min at 4°C, snap-frozen, and thawed, to accomplish complete cell lysis. Lysates were centrifuged for 10 min at 10,000 rpm and 4°C to pellet cellular debris, and the supernatants were collected and supplemented with 350 units of RNase T1 and 1.5 units of RNase A. RNA digestion was performed for 30 min at 37°C with agitation. For DNase treatment, cells were lysed in 1× DNase buffer supplemented with 0.5% NP-40. After completing cell lysis and lysate pre-clearing (see above), samples were incubated with 2 μL of DNase I (Promega) for 1 h at 37°C.

### Additional technical recommendations

Positive (e.g., MOV10 or hnRNPC) and negative controls (eGFP and parental cell lines) are well suited references to monitor the performance of the dual fluorescence RNA-binding assay as well as to define the dynamic range of the assay. RNase treatment can be used as an additional control.

When working with RBPs with low cross-linking efficiency, the UV dosage can be optimized. Whereas RNA is damaged by exposure to high dosages of 254-nm UV irradiation, 365-nm UV light does not promote RNA breaks. Thus, 365-nm UV irradiation can be increased up to 0.9 J/cm^2^ to maximize 4-SU-mediated protein–RNA covalent bonds.

After immunoprecipitation, we suggest to exclude all samples in which GFP-derived fluorescence does not exceed the background (defined by parental cell line) by at least threefold. When the protein of interest is not well expressed, the number of cells used to prepare the lysate can be increased using the same experimental settings as described above. Three biological replicates and three technical replicates typically suffice for robust statistical analyses.

### Interactome capture

Two 500-cm^2^ dishes with 40%–50% cell confluence per condition are induced overnight and processed as described (Castello et al. 2013b). Cells are lysed into 5 mL lysis buffer (20 mM Tris HCl pH 7.5, 500 mM LiCl, 0.5% LiDS [wt/v, stock 10%], 1 mM EDTA, 5 mM DTT). Samples were homogenized, and processed following the small scale settings of the interactome capture protocol (Castello et al. 2013b). Data acquisition was performed using a TECAN Safire II microplate reader with the following settings: excitation 480 nm, emission 515 nm.

### Western blotting and silver staining

eGFP- or YFP-fused proteins immunoprecipited with GBP were analyzed by silver staining, according to standard protocols, and by Western blotting using a rat antibody against GFP (3H9, Chromotek) following the manufacturer's recommendations.

## SUPPLEMENTAL MATERIAL

Supplemental material is available for this article.

## COMPETING INTEREST STATEMENT

Ulrich Rothbauer is a shareholder of ChromoTek GmbH, Martinsried.
